# Ectopic Expressions of the *GhLETM1* Gene Reveal Sensitive Dose Effects on Precise Stamen Development and Male Fertility in Cotton

**DOI:** 10.3390/genes11070772

**Published:** 2020-07-09

**Authors:** Li Zhang, Yao Zhang, Yijie Fan, Haixia Guo, Huihui Guo, Jianfei Wu, Hongmei Wang, Yunlei Zhao, Xin Lian, Zhongyuan Gou, Yuxiao Sun, Congcong Zheng, Cuixia Chen, Fanchang Zeng

**Affiliations:** 1 State Key Laboratory of Crop Biology, College of Agronomy, Shandong Agricultural University, Tai’an 271018, China; 15610418001@163.com (L.Z.); yaozhang188@163.com (Y.Z.); yjfan@sdau.edu.cn (Y.F.); diya_haixiaguo@163.com (H.G.); hhguo@sdau.edu.cn (H.G.); jfwu@sdau.edu.cn (J.W.); xlian017@163.com (X.L.); g823125157@163.com (Z.G.); 13785817706@163.com (Y.S.); zccylxq@163.com (C.Z.); cxchen@sdau.edu.cn (C.C.); 2 State Key Laboratory of Cotton Biology, Cotton Research Institute, Chinese Academy of Agricultural Sciences, Anyang 455000, China; aywhm@163.com (H.W.); zhaoyunlei@caas.cn (Y.Z.)

**Keywords:** cotton biotechnology, male sterility, shortened filaments, indehiscent anthers, pollen abortion, gene expression regulation

## Abstract

The homologous leucine zipper/EF-hand-containing transmembranes (LETMs) are highly conserved across a broad range of eukaryotic organisms. The LETM functional characteristics involved in biological process have been identified primarily in animals, but little is known about the LETM biological function mode in plants. Based on the results of the current investigation, the *GhLETM1* gene crucially affects filament elongation and anther dehiscence of the stamen in cotton. Both excessive and lower expression of the *GhLETM1* gene lead to defective stamen development, resulting in shortened filaments and indehiscent anthers with pollen abortion. The results also showed that the phenotype of the shortened filaments was negatively correlated with anther defects in the seesaw model under the ectopic expression of *GhLETM1*. Moreover, our results notably indicated that the gene requires accurate expression and exhibits a sensitive dose effect for its proper function. This report has important fundamental and practical significance in crop science, and has crucial prospects for genetic engineering of new cytoplasmic male sterility lines and breeding of crop hybrid varieties.

## 1. Introduction

Using cytoplasmic male sterile lines to cultivate hybrid varieties in crops can produce great economic and social benefits. Therefore, it is of great theoretical and practical significant to mine new genes related to fertility and develop male sterility germplasm innovation in plants. Based on our preliminary mutant analysis earlier, we discovered that the leucine zipper/EF-hand-containing transmembrane (LETM) gene was associated with mutant phenotype of male sterility. *LETM* encodes the protein of the inner mitochondrial membrane. LETM contains only one transmembrane helix, but it behaves as a putative transporter. The homologous LETMs have conserved sequences in lower eukaryotes, animals and plants [1]. In animals, studies have shown that *LETM* mediated the regulation of mitochondrial ATP production and biogenesis [2], and that *LETM* knockdown or overexpression robustly increases or decreases the mitochondrial Ca^2+^ levels in cells, respectively [3]. Moreover, researchers published their findings on the relationship between mitochondrial DNA and cytoplasmic male sterility (CMS) [4,5,6,7]. It is believed that the mitochondrial genome is the carrier of CMS factors. In plants, research has shown that a bZIP transcription factor can cause aberrant pollen and low fertility in rice [8]. LETM also has a leucine zipper structure similar to the bZIP transcription factor. So, based on the previous reports and our finding above, it suggests that LETM has a potentially crucial effect and strict expression dose mode in plant stamen development and male fertility. However, so far in plants, the LETM biological function mode remains unknown and largely unexplored.

## 2. Materials and Methods

### 2.1. Plant Material

The studied plant material was upland cotton (*Gossypium hirsutum* L.), genotype TM-1, which was grown in an experimental field at the South Campus of Shandong Agricultural University in Tai′an, Shandong Province, PR China. 

### 2.2. Cloning RT-PCR

Total RNA was isolated from 50 mg plant tissue with a TRI reagent according to the manufacturer’s instructions (T9424, Sigma-Aldrich, St. Louis., MO, USA). Then, 0.5 μg RNA was used for first-strand cDNA synthesis using the EasyScript ^®^One-Step gDNA Removal and cDNA Synthesis SuperMix (TRAN, Beijing, CN). Next, cDNA was used as a template to amplify full length cDNA of *GhLETM1* (Gh_A13G094600) using the following primers: forward 5′-CACCATGGCTTCAAGAGTGATCTTGCGAA-3′; reverse 5′-CTACGATCTTCCTGCTTCAGCAGTG-3′. In addition, the specific unique fragment of *GhLETM1* was amplified using the following primers: forward 5′-CACCGTTGTGTGACTGGCTGGATTTATCT-3′; reverse 5′-AATTCCAACTTTCTCCTCCTTTCTG-3′. RT-PCR was performed using an ABI 9902 (Applied Biosystems, Veriti^®^ 96-Well Thermal Cycler 9902), and the PCR program was as follows: 95 °C for 6 min, followed by 35 cycles of 95 °C for 30 s, 60 °C for 30 s, 72 °C for 30 s and finally 72 °C for 2 min. Then, amplified full length cDNA and the unique fragments were cloned and sequenced for OE (Overexpression) and RNAi (RNA interference) construction, respectively.

### 2.3. Vector Construction and Plant Transformation

To make the OE and RNAi constructs, the full length and the specific, unique cDNA region of *GhLETM1* was amplified by RT-PCR as above, and the PCR products were inserted into the plasmid vector pEarlyGate 203 and pB7GWIWG2(Ⅱ), respectively, using the LR reaction to generate the *P_35S_:GhLETM1* OE and RNAi constructs [9]. The constructs were respectively transformed into *G. hirsutum* L. TM-1 mediated by *Agrobacterium tumefaciens* (LBA4404 strain) according to previous reports [10,11,12].

### 2.4. Molecular Analysis of Transformants

Total genomic DNA was isolated from young leaves of transgenic plants and WT control using the CTAB method (Murray and Thompson, 1980), which was used for transgenic PCR screening with specific primers (forward 5′-AAGATGGACCCCCACCCACGAG-3′; reverse 5′-AATTCCAACTTTCTCCTCCTTTCTG-3′) that stretched across the pCaMV 35S sequence and *GhLETM1* sequence. Total RNA was isolated from plant stamen tissue with TRI reagent according to the manufacturer’s instructions (T9424, Sigma-Aldrich, USA), which was used for cDNA synthesis and then for qRT-PCR to assess the quantitative expression level of the native *GhLETM1* gene. The qRT-PCR was performed with an Ultra SYBR Mixture (Low ROX) using an Applied Biosystems 7500 Real-Time PCR System (Thermo Fisher, Waltham, MA, USA). The PCR program was as follows: 95 °C for 10 min, followed by 40 cycles of 95 °C for 15 s and 60 °C for 1 min. The specificity of the amplified PCR products was determined by melting curve analysis (95 °C for 15 s, 60 °C for 1 min, 95 °C for 15 s and 60 °C for 15 s). *GhUB7* (DQ116411) of *G. hirsutum* L. was used as internal control to standardize the results [13]. For each gene, the qPCR assays were repeated with triplicate runs. The relative expression levels were measured using the 2^−ΔΔCt^ analysis method.

### 2.5. Statistical Analysis

Statistical analyses were performed with one-way ANOVA with a cut-off significance at *p* < 0.05 or *p* < 0.01 to evaluate the expression differences among the transgenic lines.

## 3. Results and Discussion

**Figure 1:**
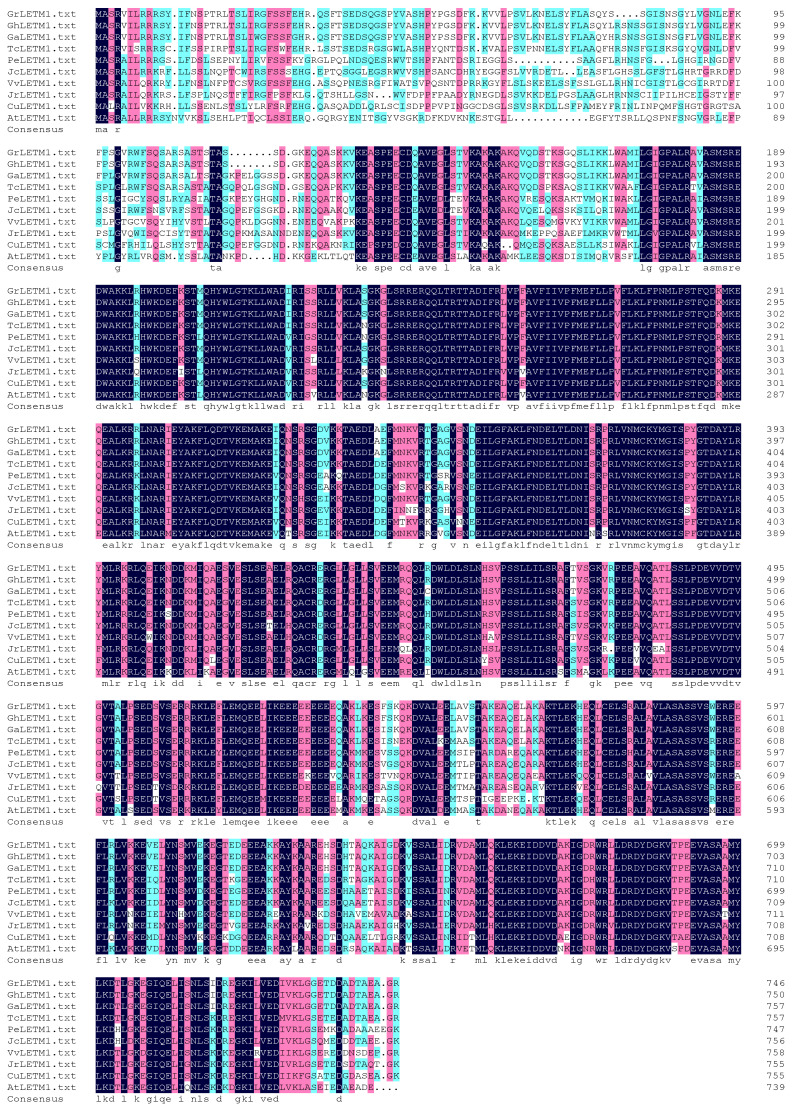
Sequence alignment and comparative analysis of LETM1 among different plants based on amino acid sequences. GhLETM1: LETM1 of *Gossypium hisutum*; GaLETM1: LETM1of *Gossypium arboreum*; GrLETM1: LETM1 of *Gossypium raimondii*; TcLETM1: LETM1 of *Theobroma cacao*; VvLETM1: LETM1 of *Vitis vinifera*; JrLETM1: LETM1 of *Juglans regia*; JcLETM1: LETM1 of *Jatropha curcas*; PeLETM1: LETM1 of *Populus euphratica*; CuLETM1: LETM1 of *Cucumis*; AtLETM1: LETM1 of *Arabidopsis thaliana*.

**Figure 2:**
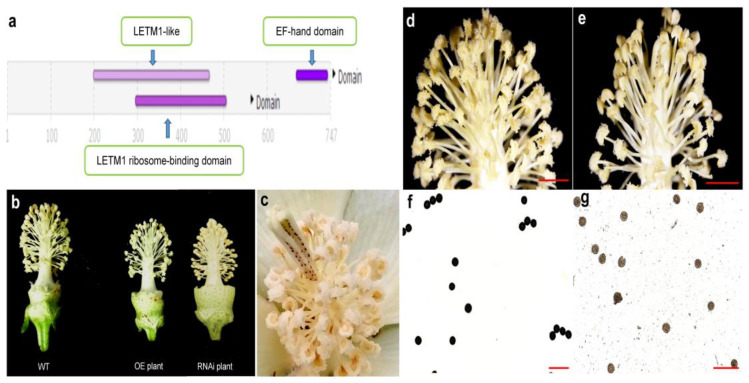
(**a**) The prediction results of the GhLETM1 protein structure. (**b**) The flower phenotype of the WT and OE/RNAi transformants. (**c**) Shortened filaments in the OE and RNAi transformants. (**d**) Normal dehiscence anthers under a stereomicroscope. Scale bars = 4 mm. (**e**) Partial indehiscence anthers under a stereomicroscope. Scale bars = 3 mm. (**f**) Normal pollen activity of the WT plant. (**g**) Pollen from indehiscence anthers are abortive without activity in the *GhLETM1* transformants. Scale bars = 500 μm.

In this project, we isolated the homologous *GhLETM1* gene in cotton as the model crop for studying stamen development and male fertility because of the big and classic flower organs. Further sequence alignment and comparative analysis of LETM among different plant species based on amino acid sequences are consistent with and extend the report above (Figure 1). GhLETM1 contains the conserved LETM1-like domain (leucine zipper/EF-hand-containing transmembrane protein 1) (Figure 2a), as has been reported in other organisms [1]. 

With the hypothesis of a strict and sensitive expression dose effect of the *LETM* gene and corresponding precise control on stamen development, the investigation purpose of the current study is to focus on the ectopic expression dose effect of *LETM* on plant stamen development and male fertility. So, we constructed *GhLETM1* OE and RNAi vectors and performed the transformation in cotton. Positive transgenic ectopic expression lines were identified in OE and RNAi, respectively. Compared to the wild-type (WT) plants, the flowers of the *GhLETM1* RNAi and OE transformants showed a size reduction (Figure 2b), with partially shortened filaments (Figure 2c) and partially indehiscent anthers (Figure 2e). Based on the length of the filaments, the phenotypes of the filaments are divided into two categories: shortened filaments and normal filaments. Based on the anther dehiscence status, the phenotypes of the anthers are divided into two categories: anther normal dehiscence and anther indehiscence. We inspected the fertility of the pollen in the OE and RNAi lines. The pollen from the control anthers were completely normal (Figure 2f), while all the pollen of anther indehiscence category presented a yellowish-brown status after staining on the contrary, which indicated that the pollen from the indehiscence anthers are defective (Figure 2g).

**Figure 3:**
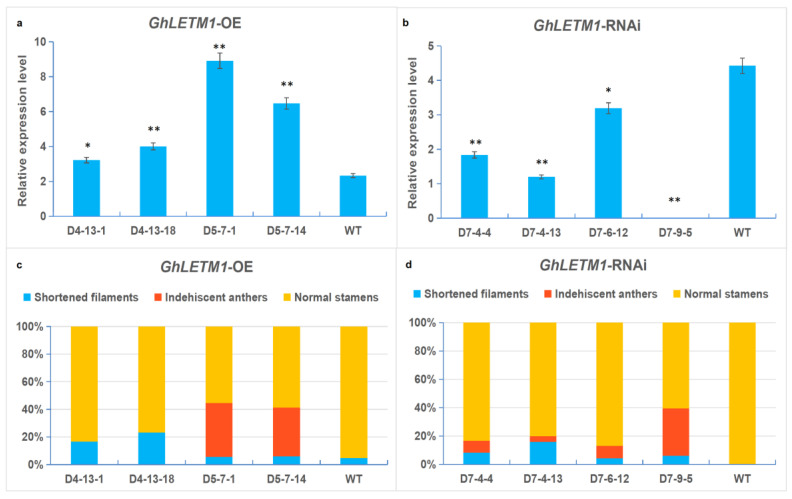
(**a**,**b**) The relative expression level analysis of the *GhLETM1* gene. Black asterisks indicate statistically significant differences between the WT plant and transgenic plants (** *p* < 0.01; * *p* < 0.05). (**a**) The relative expression level of the *GhLETM1* gene in the OE lines. (**b**) The relative expression level of the *GhLETM1* gene in the RNAi lines. (**c**,**d**) Statistics of stamen developmental abnormality in the *GhLETM1* transformants. Statistical analyses were performed based on inspection of two thousand filaments with anthers per line. (**c**) Statistical analysis of abnormal stamen development in the OE lines. (**d**) Statistical analysis of abnormal stamen development in the RNAi lines.

The expression level of *GhLETM1* in the OE and RNAi lines were further detected by quantitative real-time PCR. *GhLETM1* in the D4-13-1, D4-13-18, D5-7-1 and D5-7-14 OE transformants present significantly higher expression compared with the WT plants (Figure 3a). In the RNAi transformants, the gene expression of the D7-4-4, D7-4-13, D7-6-12 and D7-9-5 lines is obviously reduced, but with different levels (Figure 3b).

The degree of abnormality in stamen development was then investigated and analyzed in each transformant above. The D4-13-1 and D4-13-18 OE lines displayed abnormal stamen development with rates of 16% and 23%, respectively (only the shortened filaments, but all anthers, can normally dehisce). The abnormal stamen rate of the D5-7-1 line was 44% (within, shortened filaments accounted for 13.6% and anther indehiscence accounted for 86.4%) and 41% in the D5-7-14 line (14.6% shortened filaments and 85.4% anther indehiscence) (Figure 3c). For the RNAi lines, the abnormal stamen rates that appeared in the D7-4-4 lines were 15% (53.3% shortened filaments and 46.7% anther indehiscence), 20% in the D7-4-13 lines (80.0% shortened filaments and 20.0% anther indehiscence); 13% in the D7-6-12 line (30.8% shortened filaments and 69.2% anther indehiscence); and 40% in the D7-9-5 line (15.0% shortened filaments and 85.0% anther indehiscence) (Figure 3d).

Results suggested that, in the OE plants, the higher the *GhLETM1* gene, the higher the proportion of abnormal stamens; whereas in the RNAi plants, the lower the *GhLETM1* gene, the higher the proportion of abnormal stamens (Figure 3a–d). The above analysis results notably show that both the enhanced and reduced *GhLETM1* expression level were significantly correlated with degree of abnormality in stamen development. These investigations revealed that *GhLETM1* expression should be maintained at a certain level with a dose effect. Excessive and deficient expression doses would lead to abnormal stamens with shortened filaments and abortive anthers. At the same time, the results also showed that, in the lines which presented the severely defective stamens, phenotype greatly disturbed expression of *GhLETM1* (like in OE lines D4-13-1, D4-13-18, D5-7-1 and D5-7-14; RNAi lines D7-4-13 and D7-9-5). The higher proportion of shortened filaments is along with the opposite lower ratio of anther abortion and vice versa. The results indicated that the shortened filament phenotype was negatively correlated with anther abortion. It suggested that the two molecular pathways of *GhLETM1* regulation on filament shortening and anther abortion are interdependent (Figure 3c,d). 

While we detected the distinctly affected phenotypes of the stamen, we also inspected the other main organs and did not find obvious abnormal phenotypes, i.e., visible to the naked eye. There likely occurred internal trait variation and in vivo physiological characteristic changes in the ectopic expressed lines. Although our results indicate that the gene has a distinct effect on stamen development, we cannot conclude that only this developmental process is ectopically affected. 

The mitochondrial transmembrane conserved domain contained in the GhLETM1 sequence implies that GhLETM1 can play roles in plant stamen development and male fertility maintenance, likely as the unit of mitochondrial respiratory chain complexes, especially involved in respiratory chain biotransformation. However, future research is necessary to explain how the precise regulation is established and to elucidate the molecular pathway and mechanisms regulating stamen development and male fertility in plants.

A multigene family with gene duplication is a common and abundant event in plants; there are at least six LETM members that exist in the cotton (*Gossypium hirsutum* L.) genome. Still, all LETM gene members’ functions and relationships are poorly understood so far. The findings in our current study are consistent with and extend the gene balance hypothesis, which suggests that the expression levels of family gene members encoding the components of crucial molecular pathways need to be strictly maintained for precise and proper function [14]. At the same time, further experiments will be necessary for understanding the gene members’ interaction and molecular regulation network underlying stamen development and male fertility during this concerted important process.

In this study, ectopic expressions of the *GhLETM1* gene reveal a sensitive dose effect and corresponding precise control on concerted development of stamen and male fertility in cotton. This study presents significant findings on mining new genes related to male sterility in plants, which are of fundamental and practical importance in crop science, holding significant promise for its advancement in gene engineering on new male sterile lines to cultivate hybrid varieties in crop breeding. For example, we can edit gene promoters through CRISPR technology that can regulate the level of gene expression to convert the fertility of plants. In addition, we can further investigate the external optimized induction conditions for the gene expression and create environmentally induced male sterile lines. Thus, the efficiency in crop hybrid breeding can be greatly promoted using new types of male sterile lines by artificial regulation.

## 4. Conclusions

The functional mode of the potentially crucial *LETM* gene involved in the developmental process of stamen development and male fertility remains unknown and largely unexplored in plants. Based on the above ectopic expression analysis, both excessive and deficient expression of the *GhLETM1* gene lead to defective stamen development, resulting in partially shortened filaments and indehiscent anthers with pollen abortion. Further inspection also revealed that there is a sensitive ectopic expression dose effect of the *GhLETM1* gene on filament elongation and anther dehiscence in cotton. It suggests that the gene could be involved in crucial molecular pathways and need to be strictly maintained for its proper function for concerted stamen development and male fertility. The investigation results also showed that the shortened filament phenotype was negatively correlated with anther defects in the seesaw model under the ectopic expression of the *GhLETM1* gene.

## Abbreviations

Gh	*Gossypium hirsutum* L.
LETM	Leucine zipper/EF-hand-containing transmembrane protein
OE	Overexpression
RNAi	RNA interference
CMS	Cytoplasmic Male Sterility
qPCR	Quantitative Polymerase Chain Reaction
